# Temporally Arrested Breath Figure

**DOI:** 10.1021/acsami.2c05635

**Published:** 2022-06-06

**Authors:** Francis J. Dent, David Harbottle, Nicholas J. Warren, Sepideh Khodaparast

**Affiliations:** † School of Mechanical Engineering, University of Leeds, LS2 9JT Leeds, U.K.; ‡ School of Chemical and Process Engineering, University of Leeds, LS2 9JT Leeds, U.K.

**Keywords:** breath figure (BF), self-assembly, dropwise condensation, micropatterning, biomimicry, bioinspired, cicada wing

## Abstract

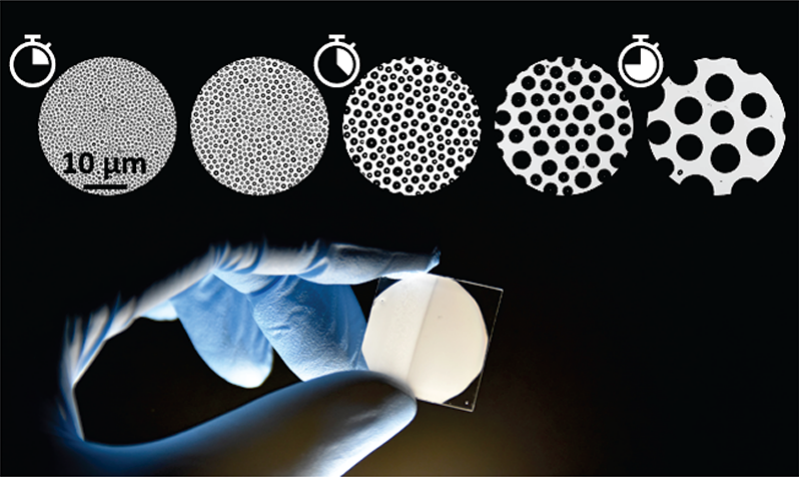

Since its original conception as a tool for manufacturing porous materials, the breath figure method (BF) and its variations have been frequently used for the fabrication of numerous micro- and nanopatterned functional surfaces. In classical BF, reliable design of the final pattern has been hindered by the dual role of solvent evaporation to initiate/control the dropwise condensation and induce polymerization, alongside the complex effects of local humidity and temperature influence. Herein, we provide a deterministic method for reliable control of BF pore diameters over a wide range of length scales and environmental conditions. To this end, we employ an adapted methodology that decouples cooling from polymerization by using a combination of initiative cooling and quasi-instantaneous UV curing to deliberately arrest the desired BF patterns in time. Through in situ real-time optical microscopy analysis of the condensation kinetics, we demonstrate that an analytically predictable self-similar regime is the predominant arrangement from early to late times *O*(10–100 s), when high-density condensation nucleation is initially achieved on the polymer films. In this regime, the temporal growth of condensation droplets follows a unified power law of *D* ∝ *t*. Identification and quantitative characterization of the scale-invariant self-similar BF regime allow fabrication of programmed pore size, ranging from hundreds of nanometers to tens of micrometers, at high surface coverage of around 40%. Finally, we show that temporal arresting of BF patterns can be further extended for selective surface patterning and/or pore size modulation by spatially masking the UV curing illumination source. Our findings bridge the gap between fundamental knowledge of dropwise condensation and applied breath figure patterning techniques, thus enabling mechanistic design and fabrication of porous materials and interfaces.

## I Introduction

**Figure 1:**
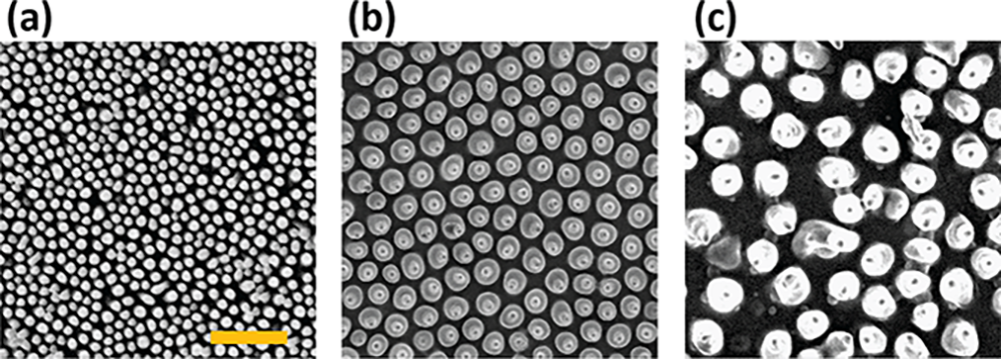
Scanning electron microscopy images of the epicuticular structure on the wings of different species of cicada: (a) *Hueschys incarnata*, (b) *Angamiana floridula*, and (c) *Gaeana loasensis*. The scale bar corresponds to 2 μm in all images.

Tailored micro and nanoscale surface topographies engender enhanced functionalities related to wetting,^1,2^ self-cleaning,^3,4^ and adhesion,^5,6^ establishing increasing application within various emerging technologies.^7−11^ Across the range of applications, biomimetic approaches aiming to replicate nature’s functionally evolved motifs have been a consistent muse for the development of novel patterned coatings.^12−15^ Among these, the striking array of circular epicuticular impressions on the wings of the cicada insect has become particularly attractive in recent years due to its supreme wetting and antimicrobial functionalities.^16−21^ The surface of cicada wings manifests self-organized hexagonally packed features of circular footprints, with diameters spanning from several hundred to thousands of nanometers, at a typically high surface coverage of 50–70% (Figure 1).^5,20,22^ Such intricate self-assembled patterns, ubiquitous in nature,^23,24^ serve not only as an end product inspiration to biomimetic surface engineering^25,26^ but as a production means in which intrinsic self-organizing mechanisms can be exploited to fabricate functional coatings in an energy efficient and scalable manner.

Over the past two decades, a variety of unconventional manufacturing processes have been explored that rely on soft matter instabilities to create self-organized patterns on the surface.^27−32^ Techniques such as microphase separation^33^ and colloidal particle templating^34^ harness fluid instabilities and capillary action to produce patterns in an inherently scalable manner. Despite offering low-cost and adaptable fabrication routes when compared to traditional micromachining/lithography techniques, soft matter surface patterning approaches still lack effective control over feature dimensions; *i.e.*, fabrication of patterns with dimensions ranging from nanometers to micrometers is often not feasible using a single patterning protocol.

The breath figure (BF) technique is an affordable soft templating approach that offers dynamic formation of hexagonally organized circular patterns, visually similar to those observed on cicada wings (Figure 1). The technique utilizes spontaneous condensation of water droplets on a polymer solution that imprint and pattern the surface as the polymer cures.^35^ To achieve BF patterns, high segment density polymers are commonly dissolved in a volatile solvent, which provides surface cooling through latent heat removal upon evaporation. In a humid environment, typically of relative humidity 50–90%, water droplets heterogeneously nucleate on the surface and grow, producing ordered droplet arrays due to Marangoni convection and thermocapillary self-organizing effects.^36,37^ During this step, the system’s viscosity increases and the imprinted droplets evaporate, resulting in a solid patterned or a porous polymer film. The adaptable sizing and large-scale templating achieved through the nonserial nature of BF offer a facile and low-cost tool for surface patterning.^38^

While the classical BF methodology and its interacting parameters have been widely discussed in several previous reviews,^37,39,40^ limited parametric studies have been completed due to the reliance upon the strict input conditions.^41^ For example, systematic control of the process is limited due to the narrow working region of relative polymer/solvent concentrations necessary to produce adequate cooling for condensation, while maintaining suitable stabilization of the templating droplets.^42^ Consequently, accurate design and full mechanistic control of the final pore size and coverage through in-line manipulation of condensation pattern remain challenging.^43−45^

Nucleation and growth of condensation droplets define the final size, morphology, and distribution of the pores formed via the BF method. Therefore, success in deterministic design and manufacturing of BF patterns relies on in-depth understanding and characterization of condensation kinetics. Fundamental investigations of dropwise condensation kinetics on dry and wet substrates have been completed by Beysens, Knobler, and co-workers.^46−50^ In these studies, deliberate control of condensation formed on solid and fluid interfaces was achieved by tuning the supersaturation level via cooling the substrate at a constant relative humidity. Although the early dropwise condensation literature provides valuable knowledge to predict kinetics of BF patterns, to date, direct adaptation of these findings for precise manufacturing of BF films has not been fully realized due to the essential coupling of pattern formation with the evaporation induced polymerization in the classical BF approach. More recently, Maniglio *et al.* showed that in situ cross-linking of UV sensitive polymers can substitute the evaporation induced polymerization.^51^ However, the proposed process still made use of solvent evaporation to induce cooling and droplet condensation. Peng *et al.* further mitigated the need for solvent evaporation through external cooling of the UV curable film by using a thermometric cooling device.^52^ Motivated by the applications in optics, this alternative BF formation approach has been mainly focused on modulating the pore morphology by adjusting the subcooling level and the working time.^53,54^ Despite successful technical developments, the proposed methods still remain largely empirical and mechanistic understandings of the impacts of the photopolymer, supersaturation conditions, and environmental parameters remain unresolved. Consequently, no reliable model yet exists for prediction of BF patterns mediated by initiative cooling in photopolymers.

**Figure 2:**
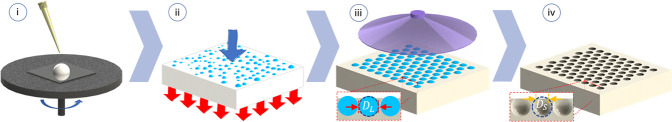
Schematic showing the main steps in the temporally arrested breath figure methodology. (i) The photosensitive polymer is pipetted onto a microscope coverslip and placed on a spin coater to form a thin film. (ii) The coverslip, with the film on top, is placed on a Peltier device equilibrated at the desired temperature to achieve subcooling, Δ*T*. Heterogeneous condensation is initiated on the top interface of the film. (iii) Droplets grow and self-assemble into a packed floating raft, before UV curing is applied at curing time, *t*_*c*_, for the duration of 5 s. (iv) Assembled water droplets evaporate as the polymer film cures, leaving the imprinted pores behind. Magnified views in (iii) and (iv) represent the maximum droplet diameter, *D*_L_, observed in the liquid precured condensation analysis, and the top surface pore diameter, *D*_S_, observed in the postcured analysis, respectively.

Here, we build upon the early analytical knowledge of dropwise condensation achieved in initiative cooling systems^49^ to further extend BF approaches for photocurable polymers^52^ and attain full control over the BF pattern dimensions. An ideal steady-state thermal condition is established to study the condensation growth rates and compare our experimental data with existing theoretical models. By readily controlling the experimental working time in a predictable manner and adding adequate handles to control the system kinetics, we gain deterministic control over BF patterns without the need for additional solvent; see the schematic of the adapted experimental approach (Figure 2). In particular, we investigate the feasibility of fabricating BF patterns with submicrometer pore size at high packing density by actively controlling nucleation density and growth rate of the condensation droplets, attaining full predictability of the final product surface properties based on the original material. Finally, we demonstrate the feasibility of spatially selective BF patterning using our adapted methodology.

## II Experimental Section

### II.1 Materials

Two single-component photocurable polymers, NOA61 and NOA63, were purchased from Norland Optical Adhesives (NOA, Norland Products Inc.) and used as received. The two thiol–ene based polymers were chosen because of their different physical properties. The dynamic viscosities of NOA61 and NOA63 are 0.3 and 2 Pa·s, respectively, as reported by the supplier. Photopolymer surface tension (SFT) in air and interfacial tension (IFT) in deionized water (Milli-Q type 1) were measured based on pendant drop analysis using a tensiometer (Theta, Biolin Scientific). For NOA61, these were calculated to be SFT = 40.5 ± 0.1 mN/m and IFT = 11.8 ± 0.2 mN/m. NOA63 exhibited relatively lower IFT in both air and water, measured as SFT = 37.0 ± 0.1 mN/m and IFT = 9.1 ± 0.8 mN/m.

### II.2 Sample Preparation

Standard borosilicate glass coverslips of 24 mm × 24 mm with thickness of 0.15 ± 0.02 mm were used as substrates for the polymer film. Coverslips were cleaned prior to use by washing with isopropanol (IPA) and deionized (DI) water before being dried in the oven overnight at 80 °C. Glass coverslips were chosen due to material compatibility with the polymers and their relatively small thickness to allow effective heat transfer. The liquid polymers were spin coated on the glass coverslips (Figure 2i) with the spin profiles set to a thin film of approximately 30 μm thickness (Figure S1). The 30 μm film is much larger than the condensation droplets considered here *O* (0.1–10 μm) to assume negligible interaction between the droplets and the substrate. The patterned films were created by initiating condensation on the wet polymer layer by placing the coated substrate on a thermoelectric Peltier cooling stage (Linkham PE120) of advertised resolution ±0.1 °C, Figure 2ii. The cooling stage was stabilized at the desired temperature before placing the coated glass coverslip at time zero.

### II.3 Condensation Growth Analysis on Liquid Films

Wet samples were analyzed precuring in real time with the Peltier stage mounted on an Olympus BX53M optical microscope (OM) equipped with a long working distance objective (Olympus LMPLFLN 50×) and a digital CMOS camera (Basler ace acA2040-90uc). The optical setup provided a nominal spatial resolution of 0.22 μm per pixel. Laboratory environmental conditions of temperature (*T*_0_) and relative humidity (RH) were monitored throughout the experiments, alongside other preparation conditions (Table 1). The subcooling level, Δ*T*, was set from the Peltier stage at the start of each experiment based on the saturation temperature at the corresponding RH and *T*_0_. The set temperature was kept constant during each experimental test lasting hundreds of seconds, with fluctuations in conditions keeping the subcooling accurate to within ±0.5 °C. Top view diametric and packing characterization of condensation droplets were performed on real-time OM images of a 444 μm × 444 μm field of view. Images were captured at the rate of 1 fps to observe the growth trends. All kinetics were recorded and analyzed starting from the initial time at which the photopolymer was exposed to the desired subcooling.

**Table 1:** Parameters and Preparation Conditions

relative humidity (%)	RH
ambient temperature (°C)	*T*_0_
subcooling (°C)	Δ*T*
time of applied curing (s)	*t*_c_
liquid droplet diameter (μm)	*D*_L_
cured pore diameter (μm)	*D*_S_
area fraction	*A*_f_
droplet number density (mm^–2^)	*N*_d_

### II.4 Photocuring and Solid Surface Analysis

Identical cooling experiments were performed under a collimated 356 nm UV flood curing system (Dymax RediCure550) to arrest the BF pattern by rapid curing of the polymer film at *t*_c_, with a 5 s exposure (Figure 2iii). Laser scanning confocal microscopy (LSCM) was performed on a Zeiss LSM800 for imaging the cured films (Figure 2iv) using a 405 nm blue laser in reflective mode due to compatibility with the long scan times and short wavelength laser. LSCM attained a lateral resolution of nominal resolution of 30 nm/pixel. Scanning electron microscopy (SEM) was also performed on cured samples using a Zeiss EVO MA15 microscope operated at 20 kV accelerating voltage.

### II.5 Quantitative Image Analysis

**Figure 3:**
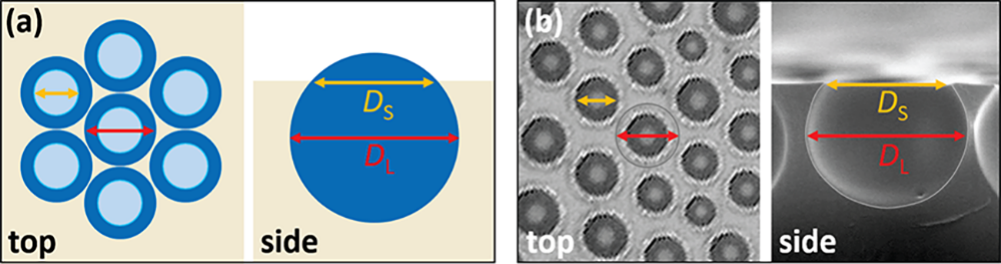
Diametric analysis of droplet patterns on the wet and cured films. (a) Schematic showing the maximum liquid droplet *D*_L_ and contact line diameter *D*_S_ from the top and side views. (b) Top (OM) and side (SEM) view images of the pores in the solid films showing the two distinct diameters.

Quantitative image characterization was performed for images acquired on both liquid and solid samples, using ImageJ^55^ and Matlab (Image Processing Toolbox, Matlab R2021b, Mathworks). The images were analyzed automatically using a circlular Hough transform identification algorithm to attain the droplet perimeters. The central portion of the frame was cropped to negate any uneven lighting, and the code was run to analyze the liquid droplet diameters, *D*_L_, in condensation analysis and solid droplet pore diameters, *D*_S_, in the precured and cured films (Figure 2iii,iv). As illustrated in Figure 3, in precured liquid films image processing quantified the maximum overall diameter of the droplet *D*_L_, due to the relative refractive indexes of water and NOA. Analysis of the cured films measured the visible pore opening on the top surface *D*_S_, corresponding to the central three phase contact line in images of precured BF films.^56^*D*_L_ is typically larger than *D*_S_ with an effectively unchanged ratio, which will be discussed later. Area fraction and number density of the droplets were calculated based on the relationships *A*_f_ =  and *N*_d_ = *n*/*A*, respectively, where *n* is the number of droplets and *A* is the size of the analyzed section on the field of view. Diameters of the droplets *D*_L_ and pores *D*_S_ were used to obtain the information on liquid and solid patterned films, respectively. Similarly, size distributions were quantified in images of both liquid and solid samples. Section S2 in the Supporting Information describes the image processing protocol and error analysis in more detail.

## III Results and Discussion

In this section, we present and discuss the quantitative data obtained by real-time analysis of condensation droplet kinetics and the postcured patterns achieved via UV exposure. For each test, independent experiments were run three times with an average taken across the repeats. Condensation analysis data on liquid films were further averaged across 15 s sampling intervals, while cured samples were analyzed in several places per sample. The trends observed in the liquid film growth are analyzed before discussion of pattern modulation control through environmental condition variation. The resultant solid patterns are compared to the liquid data sets with consideration for future applications of temporally arrested control.

### III.1 Kinetics of Heterogeneous Dropwise Condensation on Liquid Films

Monitoring the temporal evolution of condensation droplets provides valuable insight into the physical mechanisms governing their growth and spatial organization. In general, the growth of condensation droplets on a flat surface at fixed subcooling can be classified into two main regimes:^57^ (1) initial diffusion-limited growth of isolated droplets and (2) coalescence-dominated growth of highly packed droplets. In the first regime, isolated water droplets grow from individual nucleation sites without significant interactions with the neighboring droplets. Under quiescent condition, *i.e*., at constant temperature and with no significant air flow, the volume of the droplet increases proportionally to time, *V* ∝ *t*, thus leading to the relationship *D*_L_ ∝ *t*^1/3^ when assuming a constant flux of water molecules condensing onto the surface. The second growth regime occurs when the area fraction of water droplets increases to a point (typically beyond 30%)^58^ where growth through coalescence between neighboring droplets becomes the dominating regime. This regime is marked by universal pattern characteristics manifesting in a surface coverage plateau where growth is self-similar in time; coalescence of droplets leads to conservation of mass and volume but not diameter due to growth in the third dimension. The droplet growth is significantly accelerated in this regime leading to a 3-fold increase in the power-law exponent to unity, *D*_L_ ∝ *t*, for the case of three-dimensional droplet growth on a liquid film.

**Figure 4:**
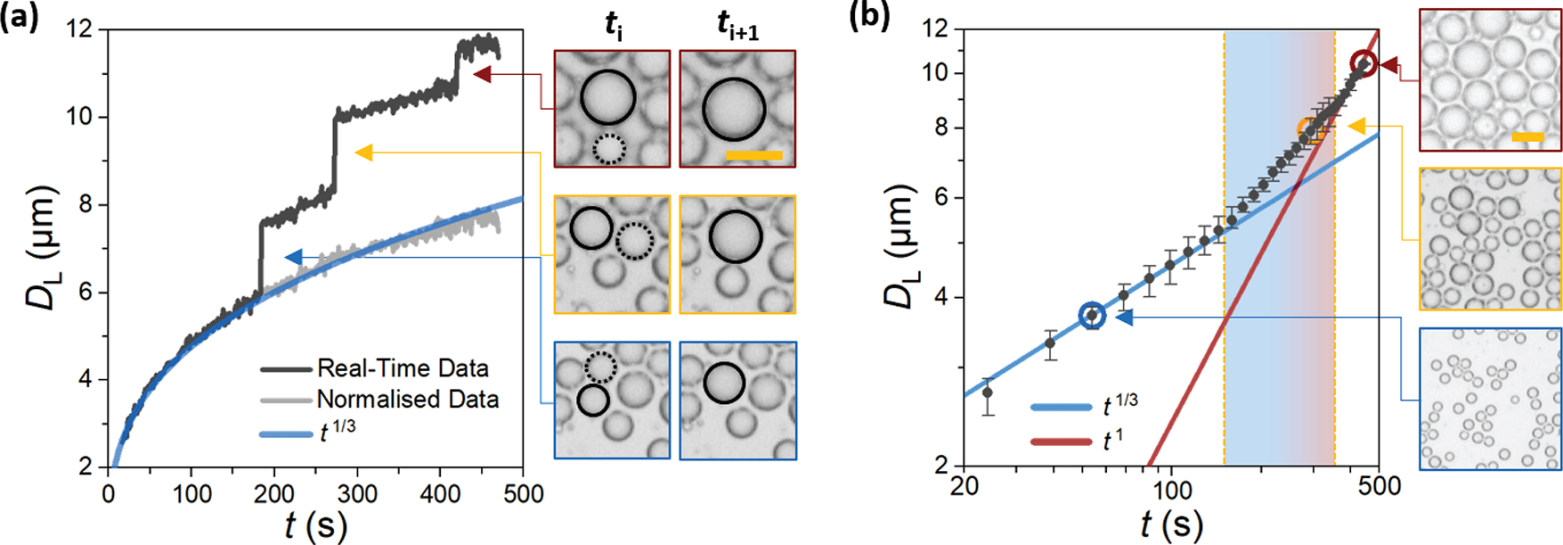
Temporal growth of condensation droplets on NOA61 recorded at RH = 26%, *T*_0_ = 24 °C, Δ*T* = 14 °C. (a) OM data for a single droplet on NOA61 growing with respect to time, showing both the coalescence-negated interpolation data and the overlaid power law trend exponent of 1/3. Overlaid images demonstrate the observed pre- and postcoalescence frames between the measured droplet (solid circle) and incident droplet (dotted circle). (b) OM data of average droplet diameter on NOA61 obtained by analyzing the full image plotted on a log–log scale. Error bars indicate the mean diametric range across the averaged 15 s data sample for the three repeats. The blue and red solid lines represent power laws describing the growth in initial diffusion-limited growth and the self-similar coalescence dominated regimes, respectively. The highlighted region indicates the transitional regime where the power law exponent increases from 1/3 to 1. The scale bars correspond to 10 μm in all images.

In order to investigate the existence of the two distinct analytically predicted growth regimes from both qualitative and quantitative perspectives, we first performed condensation experiments on NOA61. Due to the relatively larger interfacial tension with water, NOA61 provided a smaller initial nucleation density, thus allowing accurate droplet growth analysis, especially in the initial diffusion-limited regime; see Video S1. Figure 4a shows the real-time data of a single droplet throughout the analysis period. Prior to any coalescence events (*t* ≲ 200 s), the temporal growth of individual droplets is well predicted by a power law with exponent of 1/3.^46^ Sporadic jumps in diameter are observed due to occasional droplet coalescence. Subtracting the rapid droplet diameter increase effectively eliminates the coalescence jump, yielding a continuous growth that follows the original power law (*D*_L_ ∝ *t*^1/3^). This is further observed in analysis of average droplet diameter in the full frame at early time scales, *t* < 150 s, where negligible interactions occur between droplets; the data points follow the 1/3 power law with an adjusted *R*^2^ coefficient of 0.99 indicating a reliable fit in Figure 4b. Beyond this time, the growth rate increases in a transitional regime as the tighter droplet packing leads to more frequent coalescence events between neighboring droplets.^47^ The temporal growth of the droplet at *t* > 350 s tends to a power law with exponent of unity, demonstrating coalescent-dominating growth manifested by the large droplet number density (see top-right image in Figure 4b).

Due to the relatively dispersed initial droplet nucleation on NOA61, the area coverage does not reach the maximum packing until late experimental working times, by which point the droplets are relatively large. This will likely limit the pore diameter in the final BF patterns to around 10 μm and larger. As the barrier to heterogeneous nucleation is the formation of the new interfaces on the film, the lower IFT of NOA63 makes it more thermodynamically favorable for water droplets to nucleate, relative to NOA61.^59^

**Figure 5:**
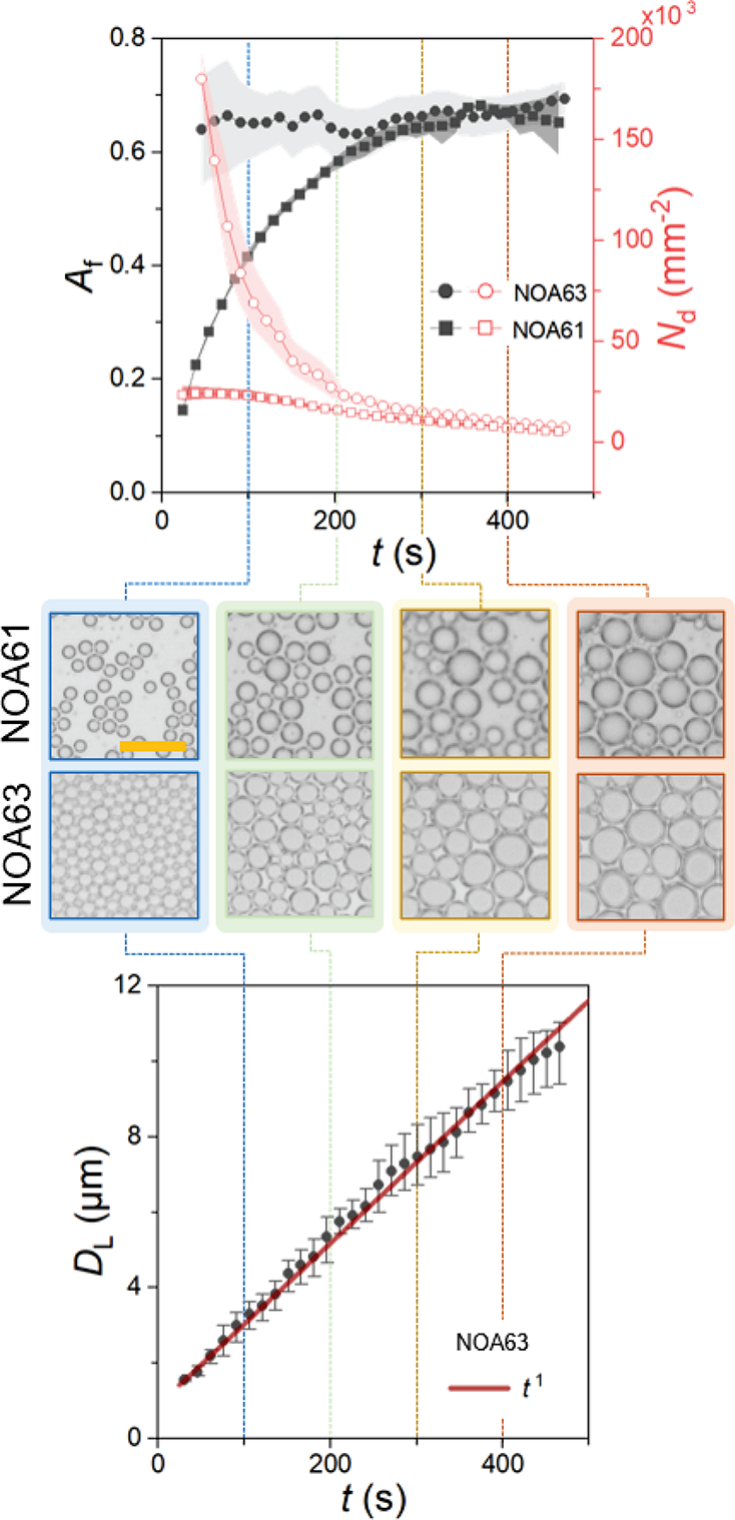
Area fraction *A*_f_ and number density *N*_d_ of condensation droplets calculated for NOA61 and NOA63. The shaded error bands represent the range in mean data across the three repeats. Representative OM images of the droplets on NOA61 and NOA63 are acquired at corresponding discrete times. The scale bar corresponds to 20 μm in all images. The diametric growth data and overlaid *D* ∝ *t* trend show the extension of the power law from early to later times for NOA63.

By quantifying the area coverage and number density of the droplets, it is expected that droplet growth on NOA63 falls in the coalescence-dominated regime from early times, where the self-similarity of droplet packing results in an effectively constant area coverage of *A*_f_ ≈ 0.66 (Figure 5). This is clearly evident in the bottom panel in Figure 5 demonstrating the diametric growth power law where the unity exponent is conserved throughout the analysis period. Because of the larger number of condensation nucleation sites on NOA63, droplets are generally smaller than those observed on NOA61 at similar conditions. Droplet growth on NOA63 is predominantly driven by coalescence, thus the diametric variation relative to the mean value in a single frame can be as large as 20%, compared to the approximate 12% variation observed in growth of isolated droplets on NOA61.

The maximum observed packing corresponds to over 70% of the theoretical packing limit in a 2D plane (around *A*_f_ ≈ 0.9), complimenting earlier studies^46^ and suggesting this is near optimal packing given the viscous liquid boundaries that stabilize/encapsulate the droplets. Droplet coalescence at such large initial number density *N*_d_ yields a quick decrease due to the large number of coalescence occurrences within the frame of view. Conversely, the distinct diffusion-limited and coalescence-dominated regimes of droplet growth observed in NOA61 (Figure 4b) leads to an initially larger increase in area fraction as droplets grow without interactions/coalescence and to a smaller decreasing rate of *N*_d_ for the same reason (see comparisons in Figure 5). At later times (*t* > 350*s*), both *A*_f_ and *N*_d_ on NOA61 films reach similar values as those found for NOA63 during the observable coalescence dominated stage, demonstrating convergence to the self-similar growth regime. Videos S1 and S2 in the Supporting Information show examples of droplet growth data for NOA61 and NOA63, respectively.

### III.2 Environmental Parameters

The self-similar growth of BF patterns on NOA63 that extends from early time (tens of seconds depending on the environmental conditions) to later time (hundreds of seconds and beyond) suggests that exposure to the UV light will allow arresting patterns of well-predicted pore size at high surface coverage using this material. Environmental parameters, however, mediate the effective patterning growth resulting from the condensation rate and thus need to be further controlled. In this adapted method, the supplemental handle of substrate temperature control replaces the indirect handle of empirical polymer solution design (polymer type, solvent, concentration) in classical BF.

**Figure 6:**
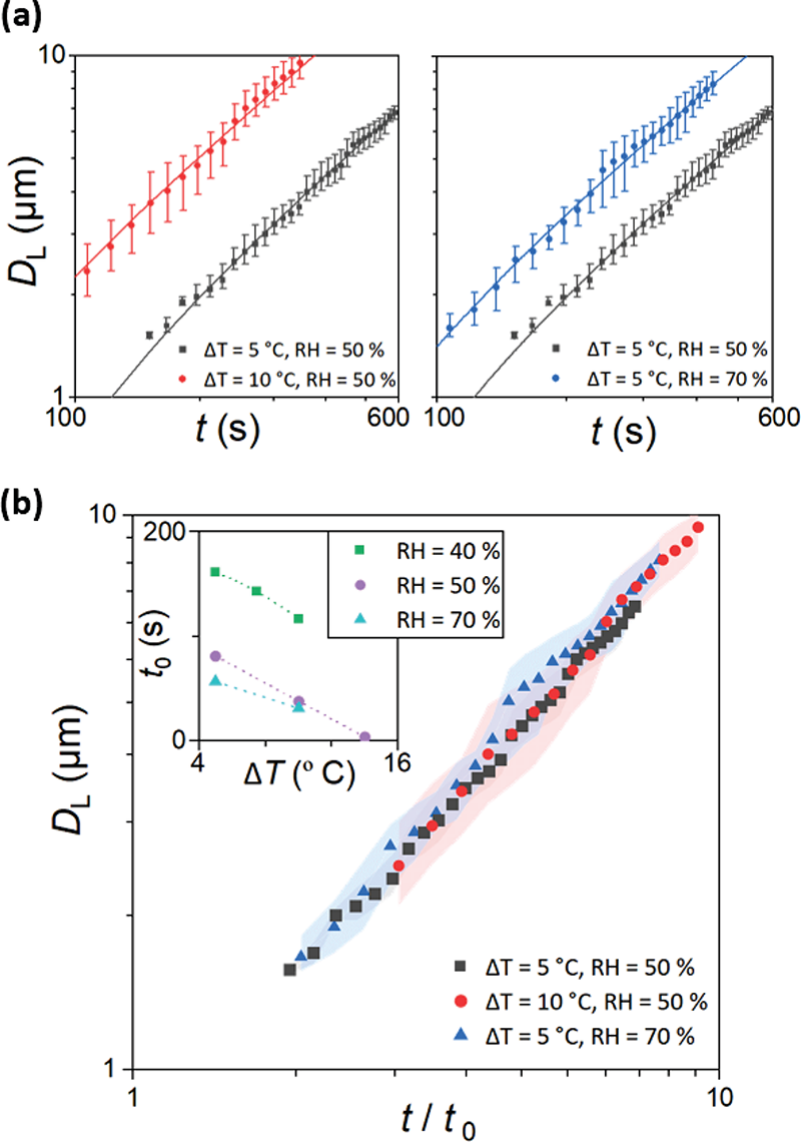
Impact of RH and Δ*T* on temporal growth of templating droplets on NOA63. (a) RH was kept constant at 50% for subcooling levels of Δ*T* = 5 °C and Δ*T* = 10° (left). For Δ*T* = 5 °C, the experiments were repeated at RH of 50% and 70% (right). Both graphs have fitted trend lines of *D* ∝ *t*. (b) Growth of droplet diameter can be collapsed onto a single power law using the dimensionless time as the new variable. *t*_0_ is an arbitrary initiation time at which average droplet diameters reach 500 nm. Inset graph demonstrates the evolution of *t*_0_ vs subcooling level at different relative humidity. The shaded error bands represent the range in mean data across the three repeats.

The growth rate of the droplet diameter is proportional to the concentration gradient and diffusion coefficient of water molecules in the vicinity of the substrate and the difference in the saturation vapor pressure.^45,57^ At temperatures close to 20 °C, the saturation pressure difference Δ*P*_s_ is proportional to Δ*T*_s_^0.8^,^57,59^ therefore, increasing the subcooling level at constant relative humidity will increase growth rate of the droplet diameter (Figure 6a, left panel). Additionally, the concentration gradient of water molecules can be controlled by modifying the relative humidity and thus the water content of the surrounding environment (Figure 6a, right panel). At a given time, the slower growth results in smaller droplet diameter variation arising from coalescent effects; this is manifested by the smaller error bars in Figure 6a for Δ*T* = 5 °C at RH = 50%. Further, due to the curing irradiation time interval, by slowing down the rate of droplet growth, smaller dimensions in the final BF patterns can be accessed, as observed in Figure S4.

Modulation of RH is commonly achieved through control of humidified gas flow, thus increasing the system complexity and causing deviation from the theoretical predictions due to hydrodynamic and thermal effects.^58^ Therefore, regulation of the substrate temperature at a fixed RH practically attains more predictable patterning across ambient and low humidity conditions.

The growth trends of templating droplets remain unchanged, following *D*_L_ ∝ *t* for all conditions, indicating that the coalescent-dominated growth regime is upheld throughout, even in the less optimal conditions (less subcooling and low RH). Due to the self-similar nature of the growth regime evident in Figure 6a, the data can be collapsed onto one single curve by compensating for the faster growth rates observed at higher subcooling and relative humidity. To this end, we define a new variable as the dimensionless time *t*/*t*_0_, where *t*_0_ is an arbitrary initiation time at which average droplet diameters reach 500 nm. The inset plot in Figure 6b demonstrates the relationship between *t*_0_ and the actively controlled subcooling level Δ*T* at different relative humidity values. As expected, the initiation time decreases as condensation is enhanced at larger subcooling and higher relative humidity. By plotting of the droplet diameters obtained at various environmental conditions (subcooling and relative humidity) versus the new dimensionless *t*/*t*_0_, all data are predictable by a new single power law *D*_L_ ∝ *t*/*t*_0_, showing that growth trends remain invariant to input environmental conditions (Figure 6b).

### III.3 Temporally Arrested BF Patterns on Solid Films

**Figure 7:**
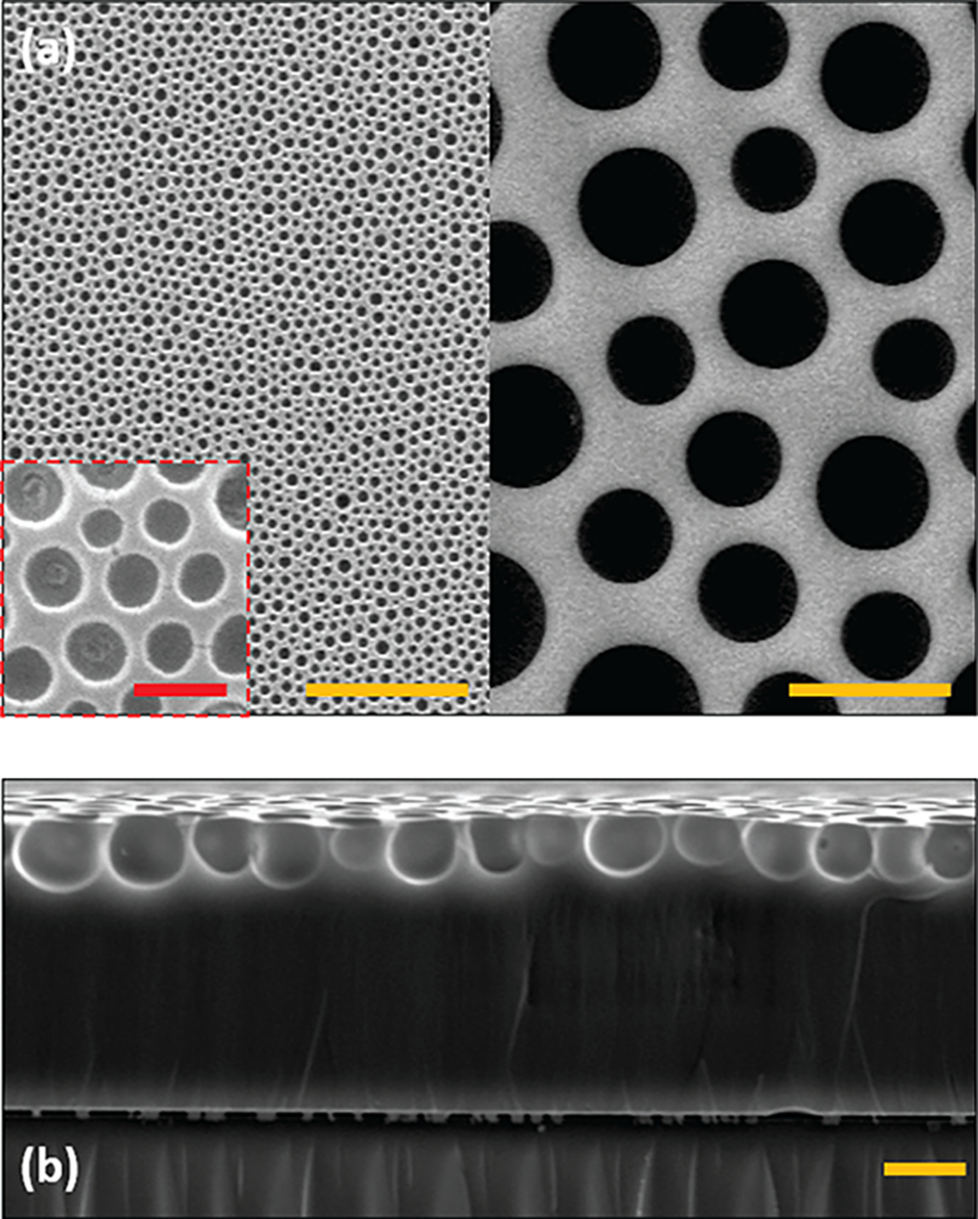
SEM images of arrested BF patterns created at RH = 63%, *T*_0_ = 22 °C, and Δ*T* = 10 °C on NOA63 films. (a) Cropped views of samples cured at *t*_c_ = 20 s (left) and *t*_c_ = 180 s (right). The red scale bar in the magnified inset corresponds to 1 μm. (b) Cross-sectional view of the pattern morphology showing the cured patterned polymer on top of the glass substrate. The dark yellow scale bars correspond to 10 μm in all images except for the inset.

NOA63 condensation experiments produced optimal packing across an order of magnitude in diameters of templating droplets. To analyze the solid films, experiments were repeated with patterns arrested upon top surface exposure to UV irradiation at discrete times, *t*_c_. Figure 7 shows example SEM images of the top view and cross-sectional view of the resultant patterns. The BF patterns on the cured films appear coalescence-dominated and thus self-similar throughout, complimenting the condensation analysis. Figure 7a shows examples of temporally arrested BF patterns with pore diameters from around 0.6 μm (left) to over 6 μm (right), demonstrating the versatility of the method. The cross-section view presented in Figure 7b confirms that the droplet assembly creates a monolayer template of semisubmerged droplets. A higher limit of approximately 10 μm was kept for diameters in this work as model natural patterns rarely exceed this dimension. Further, coalescence beyond this point led to occasional submergence of liquid droplets which subsequently interfered with the monolayer structure of the porous layer (see Video S2).

**Figure 8:**
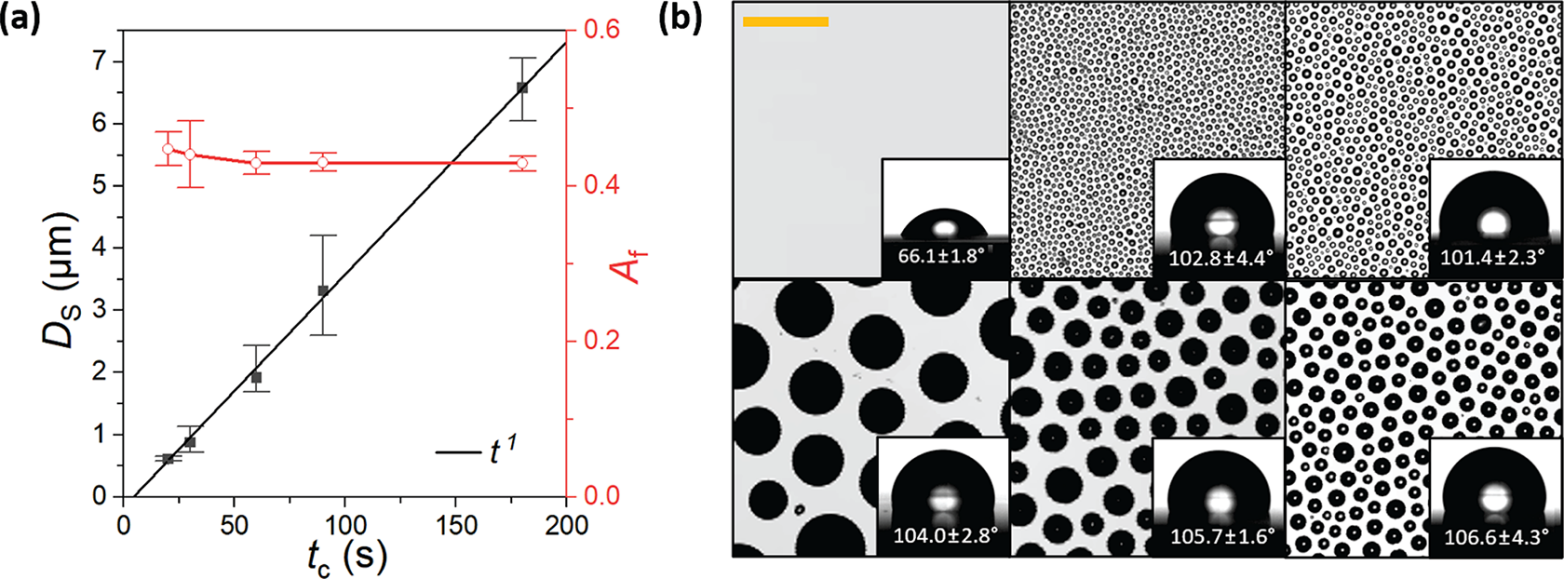
LSCM data of smooth and patterned NOA63 cured films. Area fraction, *A*_f_, and pore diameter, *D*_S_, are plotted against the respective cure times, *t*_c_, with error bars representing the range in mean data across the three repeats (a). LSCM images of the films and sessile droplet (5 μL) contact angles are shown with the standard deviation (b). Clockwise from top left, a smooth film and patterned films cured at working times of 20 s, 30 s, 60 s, 90 s, and 180 s. The scale bar corresponds to 10 μm in all images.

Side-by-side quantitative comparison between the BF patterns on the wet liquid and the cured solid films was obtained by characterizing the pores formed on cured films, using the LSCM data collected from samples cured at five different *t*_c_ values. Figure 8 shows growth relationship of *D*_S_ ∝ *t* for the arrested pore diameters, corroborating the conclusions made for the uncured wet films. Because of the higher spatial resolution of LSCM used in solid surface analysis, pores of submicrometer diameters at high packing density were observed by temporally arresting the BF on photocurable polymer films (Figure 8). Within the range of environmental conditions tested here, BF patterns with average pore diameter down to approximately 340 nm were achieved at high packing density (Figure S4). Pores of diameter as small as 100 nm are observable in SEM images (Figure S4), showing the feasibility of arresting the near-nucleation droplets in the present BF method.^59^ Additionally, comparisons of qualitative growth trends and quantitative pore diameters indicate that the effect of short burst (5 s) of UV curing on the pattern morphology is nonsignificant, with the growth power laws *D* ∝ *t* conserved throughout observed times in both solid and liquid films.

The pore area coverage, measured for the top surface openings in the cured films, plateaus at *A*_f_ ≈ 0.44, with the near-constant packing being in line with the quantitative findings of liquid droplets presented in Figure 5. The relatively smaller pore area coverage on solid surfaces compared to *A*_f_ ≈ 0.66 on wet films reflects the difference between the characteristic diameters used to calculate *A*_f_ on the liquid and solid films (see Figure 3),^56^ yielding a *D*_S_/*D*_L_ ratio of 0.67. Direct space analysis by means of Voronoi diagrams was also completed for the LSCM data sets (Figure S3) to find the average Euclidean distance between neighboring pore centers in each image, obtaining a similar value (*D*_S_/*D*_L_ = 0.72). Further analysis of cross-sectional SEM images (Figure 7b) using a small pore sample size and out of plane pores found *D*_S_/*D*_L_ = 0.7 ± 0.1. The similar ratios found corroborate the assumption that the liquid/solid *A*_f_ differences correspond to the dissimilar diametric top views between liquid (images by OM) and solid (images by LSCM and SEM) analysis and indicate that polymer shrinkage postcure is negligible. While this figure is in agreement with earlier literature reports on the droplet morphology,^46^ the ratio is ultimately set by the balance of relative interfacial energy differences, allowing potential for future shape manipulation as a result.^56^

The constant area coverage of the self-similar patterns obtained on films of effectively unchanged properties suggests that surface wettability of the patterned films is dictated solely by the wettability of the original photocurable polymers used in the process and remains unchanged as the pore diameter increases. According to the Cassie–Baxter wetting model, an average increase of around 60% in contact angle on patterned surfaces is expected relative to that on the flat cured film.^60^ Sessile water droplet analyses in Figure 8 show a similar average increase of approximately 57% in contact angle, indicating a strong increase in the hydrophobicity arising from the current BF patterning. Reported contact angles are average values of right and left contact angles for five droplets examined at different locations on films. In static conditions, no significant microscopic nonhomogeneity was observed along the contact line of the sessile droplet on the patterned surfaces (Figure S5). As expected, the contact angle data offer relatively small variance with a mean of 104 ± 3° across the averaged sample values.

**Figure 9:**
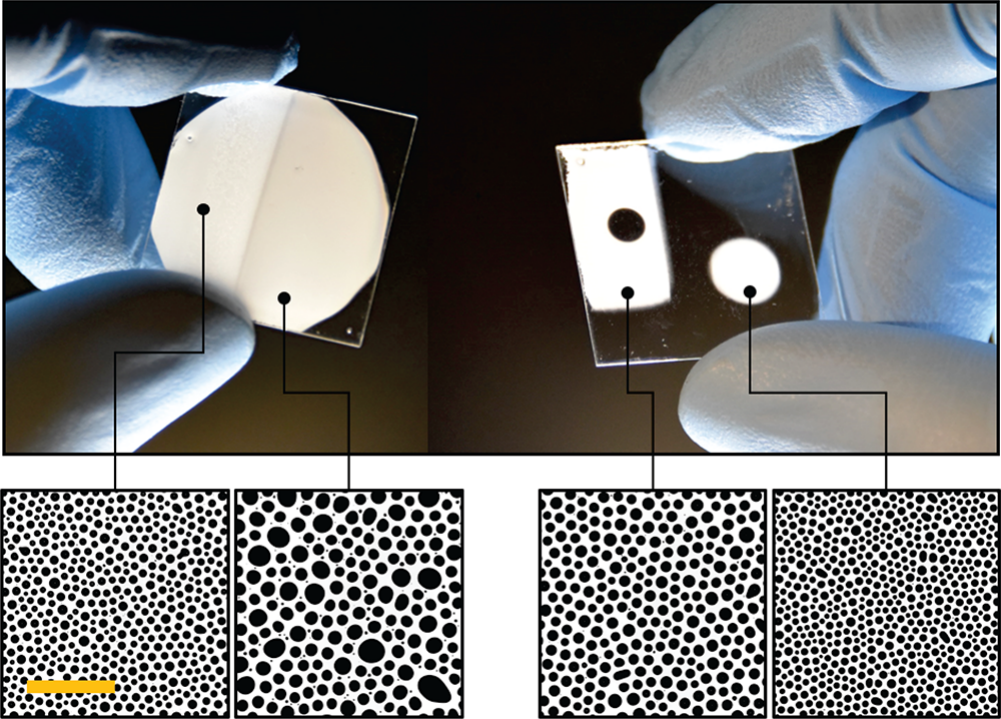
Demonstration of temporal control leading to pattern modulation capability on a single sample. The left sample shows a simple dual feature size variation. The right sample further showcases the ability to exclude patterning altogether to create specific shapes of varying feature sizes. The scale bar corresponds to 50 μm in all images.

Our findings on BF analysis of wet and cured NOA63 demonstrate that highly packed self-similar patterns can be quantitatively controlled by means of temporally arrested BF, provided that the role of environmental parameters, namely, RH and *T*_0_ can be characterized. The strict feature size control attained by the short UV exposure further provides the ability to spatially control the patterning on a single substrate. As a proof of concept, opaque UV photomasks were used to selectively cure sections of the NOA63 films at discrete times. Figure 9 shows distinct side by side patterns of small/large pores obtained by spatially UV curing the BF patterns at early/late times. Additionally, regions of pattern inclusion or exclusion with relatively sharp borders were obtained using geometrically defined UV masks.

## IV Conclusions

In summary, the present temporally modulated breath figure approach proved successful for deterministic fabrication of self-similar features, attaining average pore diameters down to around 300 nm. Our real-time quantitative experimental approach provided an ideal steady-state thermal condition to investigate the validity of the analytical heterogeneous dropwise condensation knowledge for droplet growth mechanisms and packing on liquid photocurable polymer films. Further, by replacement of the reliance upon evaporation of toxic solvents with active cooling, the determinant pore size was decoupled from the initial input material and environmental parameters, achieving greater tolerance to varied conditions relative to the classical passive-cooling breath figure methodology. Joined with the capacity to arrest these patterns via rapid and spatially selective UV photopolymerization, the current methodology opens new horizons for reliable design and fabrication of self-organized patterns via the breath figure approach. While the temporally arrested breath figure approach discussed here allows for outstanding tunability and control that are essential for a range of biomimetic surface patterning at micro- and nanoscale, further developments rely on future studies dedicated to modulation of pore/pattern morphology^61^ and tailoring of the photopolymer chemistry.^62^

## Supporting Information Available

Additional experimental details and supporting data and information on quantitative image processing protocol (PDF)
Video S1 of condensation growth on NOA61 (MP4)
Video S2 of condensation growth on NOA63 l (MP4)
