# Supplementary material for: Temporally Arrested Breath Figure
Source: ACS Appl Mater Interfaces. 2022 Jun 6;14(23):27435–43. doi: 10.1021/acsami.2c05635 (PMC9204694; doi:10.1021/acsami.2c05635)
Supplement: Supplementary file 1 — am2c05635_si_001.pdf [file am2c05635_si_001.pdf]

Supporting Information
Temporally-arrested breath figure

Francis J. Dent[1], David Harbottle[2], Nicholas J. Warren[2], and Sepideh Khodaparast[1,*]

[1]School of Mechanical Engineering, University of Leeds, LS2 9JT Leeds, UK.
[2]School of Chemical and Process Engineering, University of Leeds, LS2 9JT Leeds, UK.
[*]email: s.khodaparast@leeds.ac.uk

List of Supporting Materials

Figure S1. Spin coating calibration curves.
Figure S2. Droplet identification protocol.
Figure S3. Euclidean distance Voronoi method.
Figure S4. SEM images of NOA63.
Figure S5. Sessile droplet contact line homogeneity.
Video S1. NOA61 condensation.
Video S2. NOA63 condensation.

S1 Spin coating film thickness

Thickness of NOA films was controlled via modulating the spin coating parameters. A spin profile of 500 rpm/s acceleration and top speed of 1000 rpm and 2000 rpm was used for NOA61 and NOA63, respectively, due to the significantly different viscosities. Thickness - spin time calibration curves were produced for both NOA61 and NOA63 to attain an approximate film thickness of 30 µm (Fig. S1). The film thickness was measured through cross-sectional optical microscopy images of the cured films.

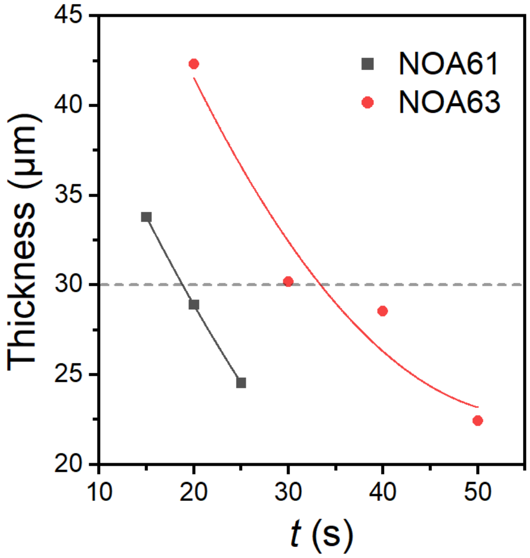

Figure S1: Spin coating calibration curve for NOA61 and NOA63. The dashed line indicates the 30 µm thickness target.

S2 Droplet identification protocol

The figure below describes the predominant thresholding regime used for both liquid and solid patterning identification. The circle identification function on Matlab based off the Circular Hough Transforms was used to detect droplet edge regions as described in the Fig. S2.

Film analysis measured the visible pore diameters in the respective liquid (D_{L}) and solid (D_{S}) pattern images. Area fraction and number density of the droplets were calculated based on the relationships $A_f = \frac{[\sum_{i=1}^{n} \pi(D_i^2/4)]}{A}$ and $N_d = \frac{n}{A}$, respectively, where n is the number of droplets and A is the size of the analysed section on the field of view. The circular identification thresholding is limited to around a 5 pixel diameter droplet size ($\approx$ 1 µm) due to the composition of square pixels. Poor contrast at small droplet and concentrated packing (early time NOA63 data) regimes thus encounter the largest error in analysis.

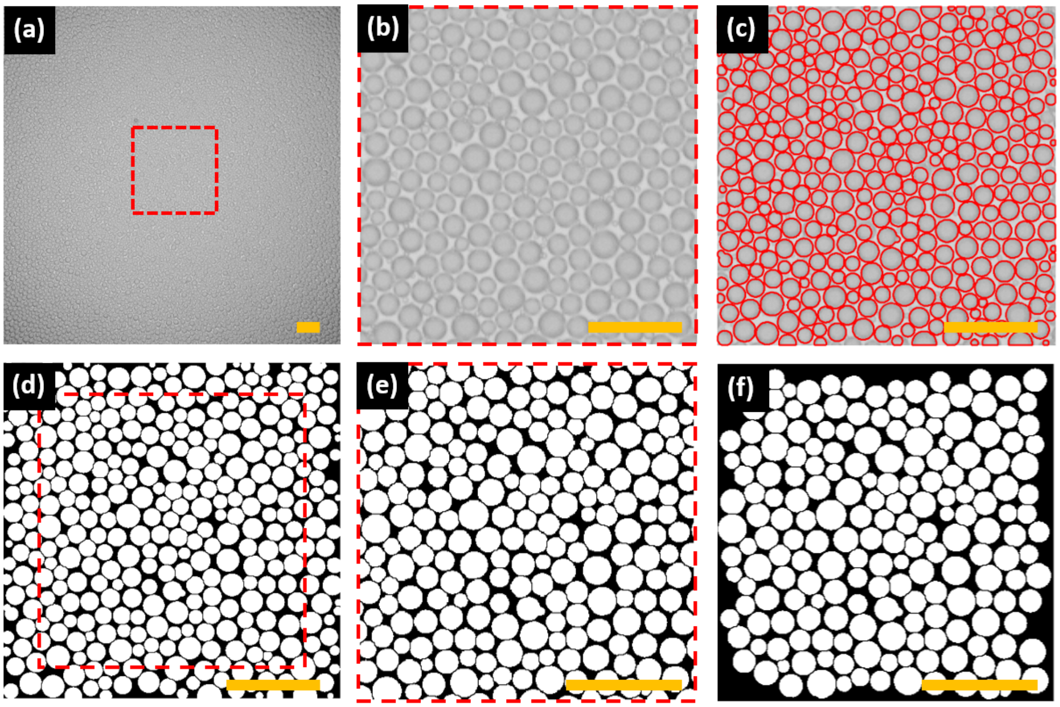

Figure S2: Droplet identification protocol used on full size microscope images (a). The central 500 × 500 pixels of the image frame is cropped (b) to perform threshold operations with the MATLAB Circular Hough Transform (CHT) function (c). The cropped view negates uneven perimeter lighting and variable focus effects whilst decreasing the computational cost. Partially occluded droplets at the frame border are not always identified (c), so the outer 10% pixel boundary of this region is further cropped (d). Analysis of the area and packing density are performed on this central 400 × 400 pixels mask and the image is saved (e). Non-complete edge droplets are excluded (f) to calculate the mean, median and standard deviation of droplet diameters. The scale bars in all panels correspond to 30 µm.

S3 Direct space analysis

Pattern anlysis on cured films was undertaken on composite laser scanning confocal images, Fig. S3(a). The same analysis regime to real-time droplet growth data was completed to threshold and create the mask viewed in Fig. S3(b). The average distance between pores was computed with Voronoi diagram analysis, Fig. S3(c), in which the distance between droplet centres was calculated by computing the euclidean distance between droplets only sharing a cell border in the Voronoi plot. This used the assumption that all neighbouring droplets share a boundary and touch at length equal to D_L. For each confocal image, the average droplet diameter D_S attained from the resultant mask was compared to our inferred average D_L value in the Voronoi diagram, attaining a D_S/D_L ratio of 0.72.

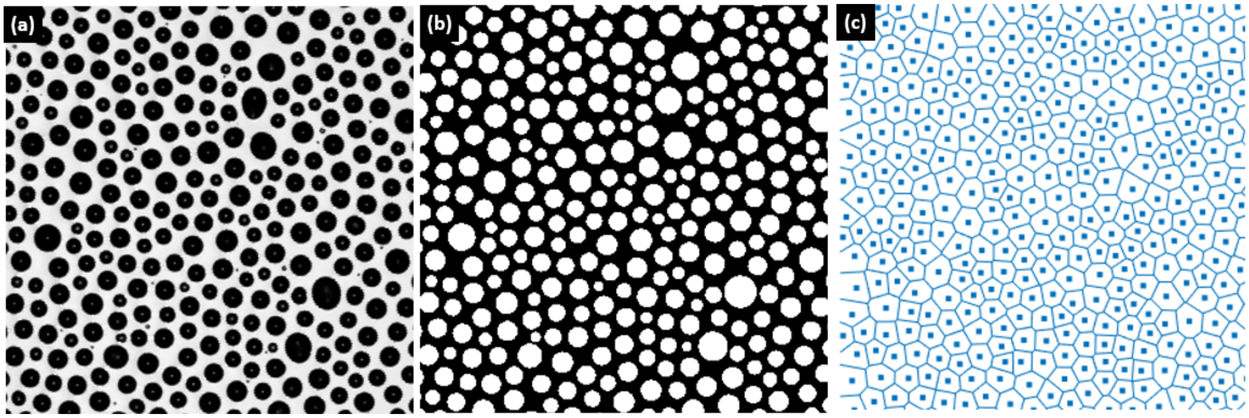

Figure S3: Composite confocal image (a), with thresholded binary mask (b) and Voronoi diagram example (c).

S4 NOA63 nanopatterned film SEM analysis

SEM images of smallest BF patterns obtained at $RH = 63\%$, $T_0 = 22$ °C and $\Delta T = 5$ °C are presented in Fig. S4. The average diameter is around 340 nm, with smallest arrested features of around 100 nm.

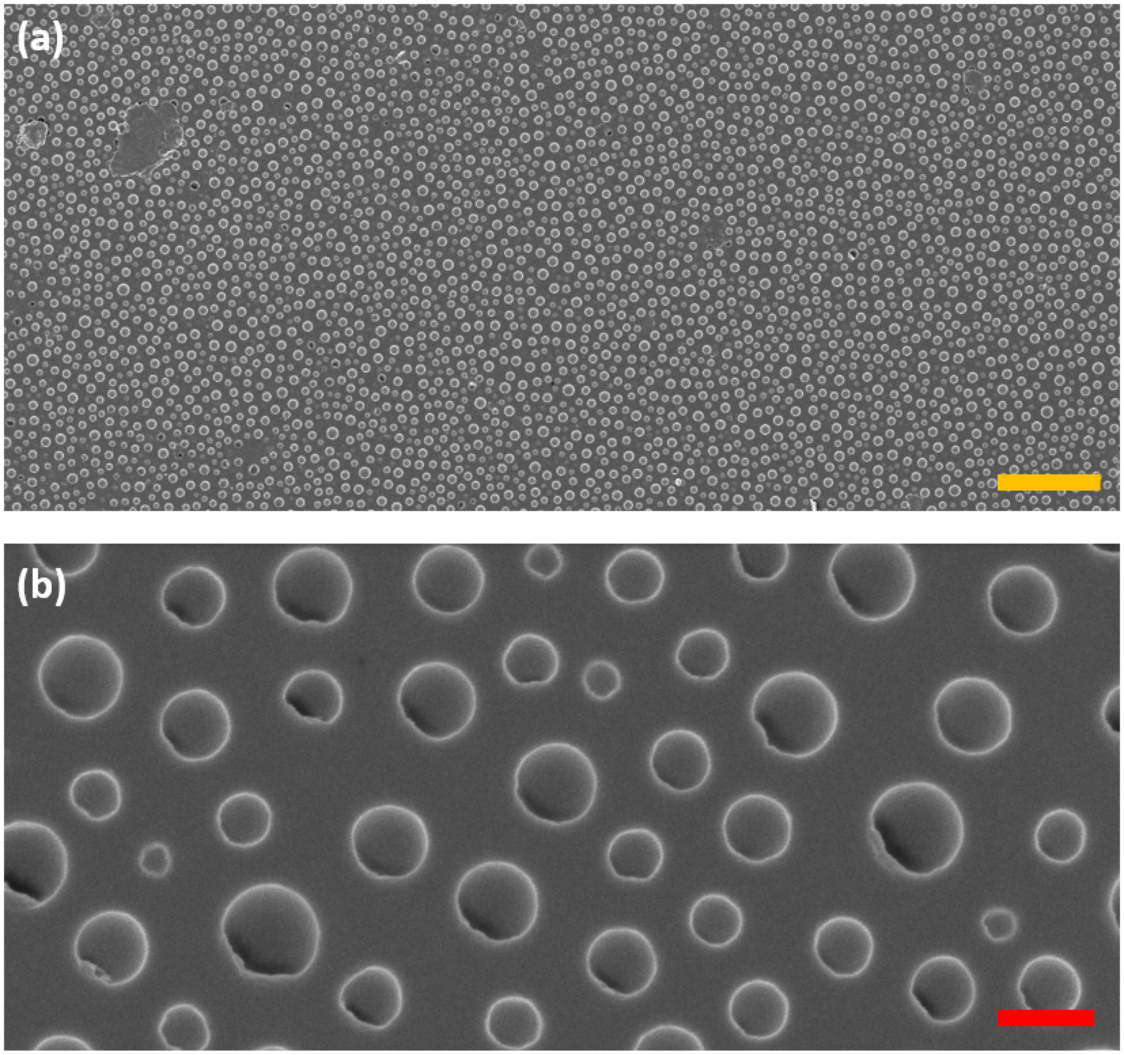

Figure S4: SEM of the smallest BF pattern achieved at $RH = 63\%$, $T_0 = 22$ °C and $\Delta T = 5$ °C. The yellow scale bar in (a) corresponds to 5 µm, and the red scale bar in magnified view presented in (b) corresponds to 500 nm.

S5 Sessile droplet contact line homogeneity

Top view optical microscopy imaging of sessile water droplets on patterned surfaces using the temporally-arrested BF methods show no significant non-homogeneity along the contact line on the solid surface, see Fig. S5.

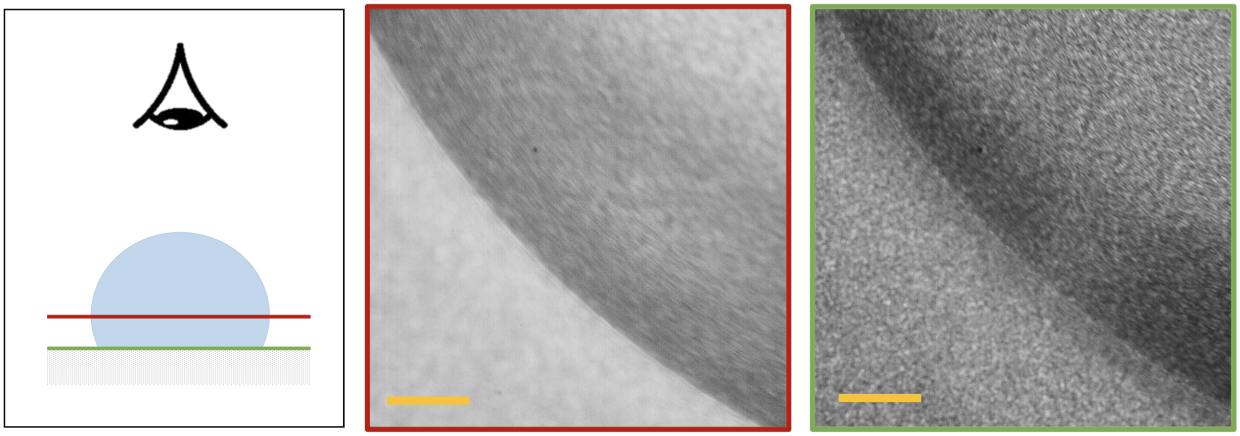

Figure S5: Top view optical microscopy image of a 3 µL water droplet on a BF patterned surface with larger pores ($D_s > 5\mu$m). The contact line on the surface (green framed image) is masked by the maximum droplet diameter (red framed image) when viewed from the top and can be imaged only through high magnification objective with a thin focal plane (high numerical aperture). The scale bar corresponds to 100 µm on both images.
